# Lee Silverman voice treatment versus standard NHS speech and language therapy versus control in Parkinson’s disease (PD COMM pilot): study protocol for a randomized controlled trial

**DOI:** 10.1186/1745-6215-15-213

**Published:** 2014-06-07

**Authors:** Catherine M Sackley, Christina H Smith, Caroline Rick, Marian C Brady, Natalie Ives, Ramilla Patel, Helen Roberts, Francis Dowling, Sue Jowett, Keith Wheatley, Smitaa Patel, Debbie Kelly, Gina Sands, Carl Clarke

**Affiliations:** 1 School of Rehabilitation Sciences, University of East Anglia, Earlham Road, Norwich NR4 7TJ, UK; 2 Division of Psychology and Language Science, Faculty of Brain Sciences, University College London, Wakefield Street, London WC1N 1PF, UK; 3 Birmingham Clinical Trials Unit (BCTU), University of Birmingham, Hospital Drive, Birmingham B15 2TT, UK; 4 Nursing, Midwifery and Allied Health Professions Research Unit, Glasgow Caledonian University, Cowcaddens Road, Glasgow G4 0BA, UK; 5 Parkinson’s UK West Midlands Regional Branch, Head office, 215 Vauxhall Bridge Road, London SW1V 1EJ, UK; 6 Faculty of Medicine, University of Southampton, University Road, Southampton SO17 1BJ, UK; 7 School of Health and Population Sciences, University of Birmingham, University Road West, Birmingham B15 2TT, UK; 8 Cancer Research UK Clinical Trials Unit (CRCTU), University of Birmingham, Hospital Drive, Birmingham B15 2TT, UK; 9 School of Clinical and Experimental Medicine, University of Birmingham, Vincent Drive, Birmingham B15 2TT, UK

**Keywords:** Parkinson’s disease, Speech and language therapy, Lee Silverman Voice Treatment, Randomized controlled trial, LSVT®

## Abstract

**Background:**

Parkinson’s disease is a common movement disorder affecting approximately 127,000 people in the UK, with an estimated two thirds having speech-related problems. Currently there is no preferred approach to speech and language therapy within the NHS and there is little evidence for the effectiveness of standard NHS therapy or Lee Silverman voice treatment. This trial aims to investigate the feasibility and acceptability of randomizing people with Parkinson’s disease-related speech or voice problems to Lee Silverman voice treatment or standard speech and language therapy compared to a no-intervention control.

**Methods/Design:**

The PD COMM pilot is a three arm, assessor-blinded, randomized controlled trial. Randomization will be computer-generated with participants randomized at a ratio of 1:1:1. Participants randomized to intervention arms will be immediately referred to the appropriate speech and language therapist. The target population are patients with a confirmed diagnosis of idiopathic Parkinson’s disease who have problems with their speech or voice. The Lee Silverman voice treatment intervention group will receive the standard regime of 16 sessions between 50 and 60 minutes in length over four weeks, with extra home practice. The standard speech and language therapy intervention group will receive a dose determined by patients’ individual needs, but not exceeding eight weeks of treatment. The control group will receive standard care with no speech and language therapy input for at least six months post-randomization. Outcomes will be assessed at baseline (pre-randomization) and post- randomization at three, six, and 12 months. The outcome measures include patient-reported voice measures, quality of life, resource use, and assessor-rated speech recordings. The recruitment aim is at least 60 participants over 21 months from 11 sites, equating to at least 20 participants in each arm of the trial. This trial is ongoing and recruitment commenced in May 2012.

**Discussion:**

This study will provide information on the feasibility and acceptability of randomizing participants to different speech and language therapies or control/deferred treatment. The findings relating to recruitment, treatment compliance, outcome measures, and effect size will inform a future phase III randomized controlled trial.

**Trial registration:**

International Standard Randomised Controlled Trial Number Register: ISRCTN75223808 registered 22 March 2012.

## Background

Parkinson’s disease (PD) is a common movement disorder affecting approximately 127,000 people in the UK alone [1]. Estimates of the prevalence of speech problems in people with PD range from between 51 and 70% [2,3] and the impact of this is known to be great, leading to increased physical and cognitive demands on the individual during conversation, reduced independence, and social withdrawal [4].

There are four main approaches to improving speech: behavioral treatment techniques, assistive aids including prosthetic and augmentative devices, and to a limited extent, medication and surgical procedures [5]. Guidelines state that speech and language therapy (SLT) should be made available for people with PD [6] but current provision is low, with a patient survey reporting that just 37% had received SLT [7].

Currently, SLT provision for people with PD who have problems with their speech or voice is based on a ’traditional’ SLT approach tailored according to individual patients’ needs, including: diaphragm breathing, pacing/rate control, word-finding strategies, and voice/articulation exercises (survey of speech and language therapists) [8]. The alternative and more recent approach is an intense prescriptive intervention called Lee Silverman voice treatment (LSVT®). The focus of LSVT® is to ‘think loud’, improving phonation and vocal loudness through improved vocal fold adduction [9]. Of the UK survey of speech and language therapy respondents, 41% had received specialist training for people with PD, with the most common being LSVT® at 67% [8].

We have performed two Cochrane reviews of SLT in PD [5,10]. The first compared the efficacy of SLT with a placebo (no intervention) and included three randomized controlled trials (RCTs) with a total of 63 PD patients [5]. Ramig *et al*. [11] (n = 29) evaluated LSVT®, whilst Robertson and Thomson [12] (n = 22) and Johnson and Pring [13] (n = 12) both investigated various forms of traditional SLT. Although improvements were reported after therapy in all three trials, the review authors concluded that due to the small number of patients examined, the low methodological quality of the trials, and the possibility of publication bias, the efficacy of SLT could not be confirmed or refuted [5]. The second review compared SLT techniques and included six trials with a total of 159 patients [10]. This review concluded that there was insufficient evidence to support the use of one form of SLT over another for PD patients [10]. Both reviews recommended that a large, methodologically sound RCT be conducted, with a follow-up period of at least six months and meaningful outcome measures [5,10]. This study aims to conduct a pilot trial to determine the feasibility and inform the design of a future phase III RCT. This pilot study will compare LSVT®, standard National Health Service (NHS) SLT, and a no-intervention control in people with PD. This pilot trial will be used to assess acceptability, recruitment, data collection procedures, compliance, outcome measures, and effect size.

The PD COMM pilot trial is designed as an assessor-blinded, multicenter RCT with three parallel groups. In line with guidance from the Medical Research Council for trials of complex interventions [14] this pilot will: assess the feasibility and acceptability of randomizing people with PD with problems of speech or voice to the LSVT® or traditional SLT interventions and to the no-intervention control; assess information on patient eligibility, recruitment, and retention rates; assess the numbers of sites, number of patients that will need to be screened, and the time required to undertake a full-scale phase III trial; assess acceptability and adherence with the LSVT® intervention; further define the dose and content of traditional SLT; determine the suitability, sensitivity, and correlation of the outcome measures; provide estimates of the effects of the interventions to inform a sample size calculation; pilot bespoke health economic evaluation questionnaire using health resource data to test the suitability of use in a future definitive trial; and assess the suitability of data collection methods.

## Methods/Design

The trial protocol and other trial documentation are available on the PD COMM website and will be updated with any modifications throughout the trial [15]. General management of the trial (including procedures for adverse events, audit, un-blinding, and data protection) will follow the University of Birmingham Primary Care Clinical Research and Trials Unit Standard Operating Procedures (SOPS).

### Recruitment

Recruitment will take place from 11 neurology and elderly care NHS clinics from a mix of urban and rural areas in the UK. The UKCRN networks will be asked to adopt the study to support recruitment. Informed consent will be obtained for all participants recruited into the study.

For a feasibility study no formal sample size calculation is required. We aim to recruit at least 60 participants over 21 months from the 11 sites (at least 20 participants in each arm of the trial) which will provide enough data to inform a future phase III trial [16].

### Inclusion criteria

The inclusion criteria are deliberately broad to allow the inclusion of a wide spectrum of typical people with PD. People with PD are eligible for this study if they: (1) have idiopathic PD defined by the UK PDS Brain Bank Criteria [17] and (2) report problems with speech or voice when asked (or their carer does).

### Exclusion criteria

The exclusion criteria for this study are as follows: (1) dementia, as defined clinically by the physician, (2) evidence of laryngeal pathology including vocal nodules, a history of vocal strain, or previous laryngeal surgery within their medical records or from discussions with client, as LSVT® is not appropriate for this group [9], (3) received SLT for PD speech or voice related problems in the past two years, based on a detectable treatment effect reported at 24 months following LSVT® [18], and (4) investigator is certain that the person with PD will not require SLT during the first six months of the trial.

The trial is 12 months duration, but participants randomized to the no-intervention group can be referred for therapy after 6 months (see below).

### Consent and randomization

**Figure 1:**
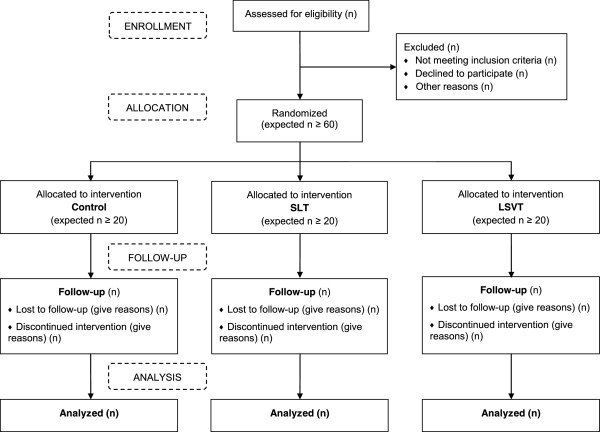
Flow diagram detailing the participant pathway through the trials LSVT®, Lee Silverman Voice Treatment; SLT, Speech and Language Therapy.

Potential participants who meet the eligibility criteria will be initially approached in their normal outpatient appointments. If interested, they will be given a patient information sheet and time to consider the trial and discuss it with friends and family. A further meeting will be arranged to answer any questions prior to informed consent being taken. Following consent, participants will complete baseline assessments prior to randomization. Participants will be informed of their treatment allocation and, if allocated therapy, be referred for the initial assessment. Baseline vocal assessments may be performed after randomization but must be completed prior to the start of therapy.After completing the baseline questionnaires, participants will be randomized between the three groups at a ratio of 1:1:1 via the Birmingham Clinical Trials Unit (BCTU) telephone randomization service (Figure 1). This secure central randomization service is available between 9 am and 5 pm weekdays and will ensure the concealment of treatment allocation. A computer-generated randomization list will be used. Participants will be informed of their treatment allocation but assessors will remain blind to treatment allocation. If allocated to an intervention arm, referral to the appropriate speech and language therapist will occur immediately following randomization. All personal information obtained for the study will be held securely and treated as strictly confidential.

### Interventions

The interventions will be delivered in outpatient and community settings. The setting will be dependent on the provision of the speech and language therapy and the needs of the individual participant. Logs summarizing the intervention will be kept by the therapist and will be used to monitor adherence and, in the case of the standard SLT intervention, to document content.

### Lee Silverman voice treatment

The intervention will replicate the dose and content prescribed by LSVT®, consisting of 16 sessions of between 50 and 60 minutes duration delivered over four weeks [9,19]. Participants will also be set 5 to 10 minutes of home practice on treatment days, and up to 30 minutes of home practice on non-treatment days [20].

LSVT® comprises maximum effort non-speech and speech drills. The non-speech drills include production of sustained ‘ah’ phonation at a single pitch and pitch glides (moving from low pitch to high pitch and vice versa on production of sustained ‘ah’). These exercises are for improving voice production and flexibility without speech. The speech drills utilize a hierarchy of speech tasks moving from single words through phrases and onto conversational speech. Each step in this hierarchy puts increased demands on the speaker and challenges the speaker to maintain maximal speech production.

### Standard speech and language therapy

The content and dose of standard SLT is poorly defined within the published literature. For this reason, the standard therapy intervention will encompass all SLT techniques that are not LSVT®. Treatment will be individualized and may include (but will not be limited to) any of the following: exercises targeting respiration, phonation, articulation [12,13], behavioral strategies to reduce prosodic abnormality [21], and the use of augmentative and alternative communication (AAC) strategies and therapeutic devices to improve functional communication [22]. The dose will be determined by the participant’s individual needs, but will not exceed eight weeks of treatment. It is most likely to reflect the median dose reported in a survey of current UK speech and language therapy practice for PD by Miller *et al*. [8] of 6 sessions averaging 45 minutes delivered over 42 days.

### Control

If allocated to the control group participants with continue with their standard care and receive no SLT input for at least six months post-randomization unless their clinician deems it to be medically necessary. The six month SLT exclusion time for this group was decided in conjunction with therapists. After six months participation in the trial people in the control arm can be referred for therapy by their usual specialist through NHS referral pathway.

### Outcome measures

Data on various outcomes measures will be collected as part of the PD COMM pilot in order to assess which outcomes are appropriate to take forward to the phase III RCT. These include both participant, therapist, and carer completed questionnaires and measures. Outcome measures will be assessed at baseline (pre-treatment), 3 months (post-randomization - to allow for any delays in referral to therapy and the varying intervention lengths), 6 months, and 12 months post-randomization (to see if any benefit is maintained). Assessment will take place at the therapy departments of participating centers and will be conducted when the participant is ‘on’ medication (if medicated). Vocal assessment of intelligibility of dysarthric speech (AIDS), loudness, and comprehension will be recorded and assessed by assessors masked to the participant’s group allocation. Participant completed questionnaires will be conducted via post.

Effectiveness of communication will be measured using the self-report Voice Handicap Index (VHI) [23]. The VHI has previously been used as an outcome measure in an extended LSVT® trial for PD [20]. Intelligibility will be assessed using AIDS [24]. Participants will be digitally recorded speaking randomly generated words and sentences from AIDS. These will then be transcribed by three blinded assessors. Vocal loudness will be measured through sound pressure level (in decibels) for a sustained vowel (’ah’) phonation, the reading of a ‘rainbow passage” (an articulation exercise including all the normal sounds of spoken English), and speaking freely during a self-chosen monologue [18]. These will be audio-recorded and assessed by a blinded assessor. A comprehensibility assessment will be made using a picture, the ‘cookie theft’ picture from the Boston diagnostic aphasia examination [25]. This will be digitally recorded and assessed by three blinded assessors. Quality of life (QoL) will be recorded through two self-report measures; the PD-specific 39 point Parkinson’s Disease Questionnaire (PDQ-39 [26] and the voice specific Voice-Related QoL scale (V-RQOL) [27]. Participation restriction related to speech and communication will be assessed using the self-report Living with Dysarthria questionnaire (LwD) [28]. The effect on carers will be measured through the self-report Parkinson’s Carers’ Quality of Life Questionnaire [29]. Screening and treatment logs will also be completed to collect data relating to acceptability of the intervention and follow-up, recruitment and retention rates, and participant adherence to treatment allocation.

Health-related quality of life will be obtained using the EuroQol 5 Dimensions (EQ-5D) [30], capability measurement using the ICEpop CAPability measure for Older people (ICECAP-O) [31,32], and resource use using a bespoke self-completion questionnaire. For time points of assessment see Table 1.

**Table 1:** Outcome measures and assessment time points

**Assessment type**	**Completed by**	**Time of administration**			
		**Baseline**	**3 months**	**6 months**	**12 months**
Hoehn and Yahr scale	Clinician	✓		✓	✓
Medication	Clinician	✓		✓	✓
AIDS	Therapist	✓	✓	✓	
Vocal Loudness	Therapist	✓	✓	✓	
Comprehensibility	Therapist	✓	✓	✓	
VHI	Participant	✓	✓	✓	✓
PDQ-39	Participant	✓	✓	✓	✓
V-RQOL	Participant	✓	✓	✓	✓
LwD	Participant	✓	✓	✓	✓
EQ5D	Participant	✓	✓	✓	✓
ICECAP-O	Participant	✓	✓	✓	✓
Resource use questionnaire	Participant		✓	✓	✓
Parkinson’s Carers’ QoL Questionnaire	Carer	✓	✓	✓	✓

Legend: Assessment of intelligibility of dysarthric speech (AIDS); Voice Handicap Index (VHI); Parkinson’s Disease Questionnaire (PDQ-39); Voice-Related QoL scale (V-RQOL); Living with Dysarthria questionnaire (LwD); EuroQol 5 Dimensions (EQ-5D); ICEpop CAPability measure for Older people (ICECAP-O).

### Ethical approval and oversight

The PD COMM pilot trial has been granted National Research Ethics Service (NRES) approval (Reference: 11/WM/0343) and has an international standard randomized controlled trial number (ISRCTN75223808).

The trial also has a joint Data Management Committee (DMC) and Trial Steering Committee (TSC) to monitor the progress and safety of the trial and ensure the protocol is adhered to. The joint TSC/DMC includes an independent chair and two further independent members.

### Safety reporting

The SLT interventions within this pilot study are considered low risk. There may be a small increased risk of vocal strain or abuse and this small risk will be clearly stated in the participant information sheet. Every effort will be made to minimize the risk of vocal strain or abuse. Speech and language therapists are trained to identify and rehabilitate vocal strain, so if present, the therapists can quickly address it. No other risks are expected to arise from taking part in the study. It is therefore reasonable to only collect targeted-treatment related adverse events and serious adverse events such as vocal strain or abuse. These will be collected on a study serious adverse event form.

### Analysis

#### Feasibility and acceptability

Data from screening logs completed by each centre will provide information on the participant screening process and will be analyzed descriptively. Reasons for non-entry into the trial will also be assessed, particularly in relation to the patient eligibility criteria and reasons for patient refusal.

Data on patients who do not complete the trial (such as withdrawals and those lost to follow-up) will also be collected throughout the trial to allow assessment of patient retention rates and reasons for non-completion of the trial. Reasons for non-completion will be analyzed descriptively.

#### Adherence to SLT interventions

LSVT® is an intensive course of treatment. In this group therapists will provide information on the mean number and duration of sessions each patient receives during the study in order to assess patient and therapist compliance with LSVT®. Patients will also be asked to document SLT homework in diaries.

Therapists administering standard NHS SLT will also be logging information on the SLT sessions providing information on number and duration of sessions and the type of therapy provided. This will give some insight into the content and fidelity of standard NHS SLT, with the mean number and duration of sessions presented, along with a descriptive analysis of the SLT content.

#### Outcome data

Data from this pilot study will also be used to inform the choice of outcome measures, provide data to inform a sample size calculation, and pilot the health economic evaluation for the full-scale trial.

Data return rates at each time point will be assessed along with data completeness of the various outcomes measures. The outcome data collected will be summarized using descriptive statistics and an exploratory analysis will be performed. Analyses will be performed using intention-to-treat. Data at each time point for each arm will be presented as means with standard deviations. The differences between the arms (LSVT® versus no intervention, NHS SLT versus no intervention, and LSVT® versus standard NHS SLT) in the means and mean change from baseline to 3, 6, and 12 months will be calculated, along with the 95% confidence intervals. This will help to determine the sensitivity of the outcome measures to change. Appropriate techniques (such as correlation methods) will be used to identify which outcome measures are closely related, which will then inform which outcome measures to take forward for use in a full-scale phase III trial. The data collected within this pilot study (estimate of the variability and treatment effect size) will help inform a sample size calculation for a full-scale trial.

#### Health economics

The trial will pilot the tools to measure resource use and outcomes that may be used in a full economic evaluation alongside a larger trial. The rates of completion and sensitivity to change will be determined.

## Discussion

Speech and language therapy is an important component of treatment for people with PD. Two recent Cochrane reviews have concluded that evidence for the efficacy of SLT and different SLT techniques is lacking and therefore well-designed RCTs are needed to address this issue [5,10]. This trial will provide important information relating to the feasibility of a definitive RCT of the effect of SLT and LSVT® in people with PD. All aspects of this trial will be scrutinized including the recruitment methods, data collection procedures, outcome measures, follow-up time points, and intervention adherence in clinical and home settings. This pilot study is a well-designed, rigorous RCT which is the largest in the field to date. It has many strengths including robust blinding and detailed approaches to the measurement and evaluation of speech, and will capture previously unconsidered information on adherence to home-based tasks. As this is a pilot trial, effectiveness will not be established. However, the data collected will be of vital importance to future clinical research and necessary to inform a phase III RCT to test the clinical and cost-effectiveness of SLT and LSVT® on speech problems in people with PD.

## Trial status

The PD COMM pilot trial is ongoing and has not completed participant recruitment which began May 2012.

## Abbreviations

AAC: Augmentative and Alternative Communication; AIDS: Assessment of intelligibility of dysarthric speech; EQ-5D: EuroQol 5 Dimensions; ICECAP-O: ICEpop CAPability measure for Older people; LSVT®: Lee Silverman Voice Treatment; LwD: Living with Dysarthria questionnaire; NHS: National Health Service; PD: Parkinson’s Disease; PDQ-39: Parkinson’s Disease Questionnaire; RCT: Randomized Controlled Trial; SLT: Speech and Language Therapy; SOPS: Standard Operating Procedures; VHI: Voice Handicap Index; V-RQOL: Voice-Related QoL scale.

## Competing interests

The authors have no competing interests.

## Authors’ contributions

CMS is the Chief Investigator. CMS, CHS, CR, MCB, NI, RP, HR, FD, SJ, KW, SP, CC contributed to conception and design, critical revision, and gave final approval of the manuscript. GS contributed to drafting the manuscript, data interpretation, and gave final approval of the manuscript. DK contributed to data interpretation, critical revision, and gave final approval of the manuscript. All authors read and approved the final manuscript.
